# A Review on the Effect of Calcium Sequestering Salts on Casein Micelles: From Model Milk Protein Systems to Processed Cheese

**DOI:** 10.3390/molecules28052085

**Published:** 2023-02-23

**Authors:** Gaurav Kr Deshwal, Laura G. Gómez-Mascaraque, Mark Fenelon, Thom Huppertz

**Affiliations:** 1 Department of Food Chemistry and Technology, Teagasc Food Research Centre, Fermoy, P61C996 Cork, Ireland; 2 Department of Agrotechnology and Food Sciences, Wageningen University, Bornse Weilanden 9, 6708 WG Wageningen, The Netherlands; 3 Dairy Technology Division, ICAR-National Dairy Research Institute, Karnal 132001, Haryana, India; 4 FrieslandCampina, Stationsplein 4, 3818 LE Amersfoort, The Netherlands

**Keywords:** cheese, citrates, phosphates, casein micelles, calcium sequestering salts

## Abstract

Phosphates and citrates are calcium sequestering salts (CSS) most commonly used in the manufacture of processed cheese, either singly or in mixtures. Caseins are the main structure forming elements in processed cheese. Calcium sequestering salts decrease the concentration of free calcium ions by sequestering calcium from the aqueous phase and dissociates the casein micelles into small clusters by altering the calcium equilibrium, thereby resulting in enhanced hydration and voluminosity of the micelles. Several researchers have studied milk protein systems such as rennet casein, milk protein concentrate, skim milk powder, and micellar casein concentrate to elucidate the influence of calcium sequestering salts on (para-)casein micelles. This review paper provides an overview of the effects of calcium sequestering salts on the properties of casein micelles and consequently the physico-chemical, textural, functional, and sensorial attributes of processed cheese. A lack of proper understanding of the mechanisms underlying the action of calcium sequestering salts on the processed cheese characteristics increases the risk of failed production, leading to the waste of resources and unacceptable sensorial, appearance, and textural attributes, which adversely affect the financial side of processors and customer expectations.

## 1. Introduction

Bovine casein micelles are polydisperse spherical particles having a mean radius of roughly 100 nm [1]. They are primarily composed of α_s1_-, α_s2_-, β-, and κ-casein in the weight ratio of ~3:0.8:3:1. Their integrity is held by colloidal calcium phosphate (CCP), constituting ~7% of the micellar dry weight [2]. The attractive forces in casein may involve hydrophobic bonds, hydrogen bonds, calcium phosphate cross-links, and electrostatic interactions, whereas repulsive interactions are basically repulsive electrostatic interactions, which are mainly affected by net casein charge [3]. CCP neutralizes the negatively charged phosphoseryl residues by cross-linking casein molecules and allowing hydrophobic interaction between caseins [4]. Since α_s1_-, α_s2_-, and β-casein contain centers of phosphorylation (at least three phosphoserine groups in sequence), they can readily bind Ca^2+^ [5]. κ-Casein typically contains only one phosphoseryl residue and is less affected by the presence of calcium [6]. Calcium sequestering salts (CSS), such as phosphates and citrates, which are commonly used in the manufacturing of processed cheese (where they are often referred to as emulsifying salts or melting salts), decrease the concentration of free calcium ions by sequestering calcium from the aqueous phase, and dissociate the casein micelles by altering calcium equilibrium, thereby resulting in the enhanced hydration and volume of the micelles [7]. Upon the addition of CSS to casein micelles, CCP is removed and the micelles dissociate. During the manufacturing of processed cheese, the interactions among the casein matrices, CSS and calcium are critical factors affecting the final functional attributes such as texture, meltability, and emulsification [8].

Processed cheese is a viscoelastic matrix consisting of cheese(s) as well as several dairy ingredients (e.g., skim milk solids, butter, anhydrous milk fat, milk, whey powder, and co-precipitates) and non-dairy ingredients and additives (e.g., stabilizers, acidifying and sweetening agents, colors and flavors), to achieve the desirable functional attributes [9]. Further key ingredients in the manufacture of processed cheese are CSS, usually added as sodium salts of monomeric or polymeric phosphates or citrates [10]. According to the US Food and Drug Administration, 13 different CSS are permitted, either singly or in combination, in processed cheese manufacture (Table 1) [11]. Calcium sequestration involves the exchange of Ca^2+^ in the casein micelles, with monovalent cations (e.g., H^+^, Na^+^ or K^+^) of the CSS [12]. The quality, textural and functional properties of processed cheese are to a large extent determined by the composition and maturity of natural cheese, the quantity and type of CSS added, and several other processing parameters. CSS also plays a significant role in the formation of the required microstructure of the final product via pH adjustment and calcium sequestration [13].

Various milk protein systems, including milk protein concentrate [3,14,15], skim milk powder [16], rennet casein [12,17], and micellar casein concentrate [18,19], have been studied to elucidate the effect of CSS on the properties of casein micelles or para-casein micelles. On the other hand, several researchers have linked the changes in properties of processed cheese with the influence of CSS on the properties of casein micelles. It is likely that certain discrepancies between the research articles vis-à-vis the influence of CSS on the properties of milk protein systems exist. This is due to inter-study differences in CSS combinations, processing parameters (e.g., time-temperature combinations, mixer type, and shearing rates), and matrix formulation (e.g., levels of moisture, protein, and pH). Regardless, these research articles provide insights about the functionality of CSS in processed cheese matrix development and its effect on rheological, functional and physicochemical attributes. Therefore, in this review, we will focus on the different types of CSS and their influence on the solubilization of casein fractions, casein dispersion, and casein hydration. The changes in the viscoelastic, textural, and functional properties of processed cheese systems are also considered in a separate section for better insights on the functionality of CSS in complex milk protein systems.

## 2. Different Types of Calcium Sequestering Salts

Calcium sequestering salts have monovalent cations (e.g., Na^+^, K^+^, and H^+^) and polyvalent anions (e.g., phosphates or citrate). The CSS most commonly used in processed cheese are divided into two categories: citrates and phosphates. Commonly used citrates are trisodium citrate (TSC) and monosodium citrate (MSC), and commonly used phosphates are disodium phosphate (DSP), tetrasodium pyrophosphate (TSPP), and sodium hexametaphosphate (SHMP) [20,21]. Citrate salts are obtained by replacing the hydrogen atom from the tribasic citric acid with suitable cations (e.g., Na^+^, K^+^), resulting in the formation of mono-, di- and trisodium citrate. Depending on the dissociation steps, citric acid may form citrates, hydrogen citrates, and dihydrogen citrates. Trisodium citrate is the most commonly used citrate CSS in the manufacturing of processed cheese [22].

**Figure 1:**
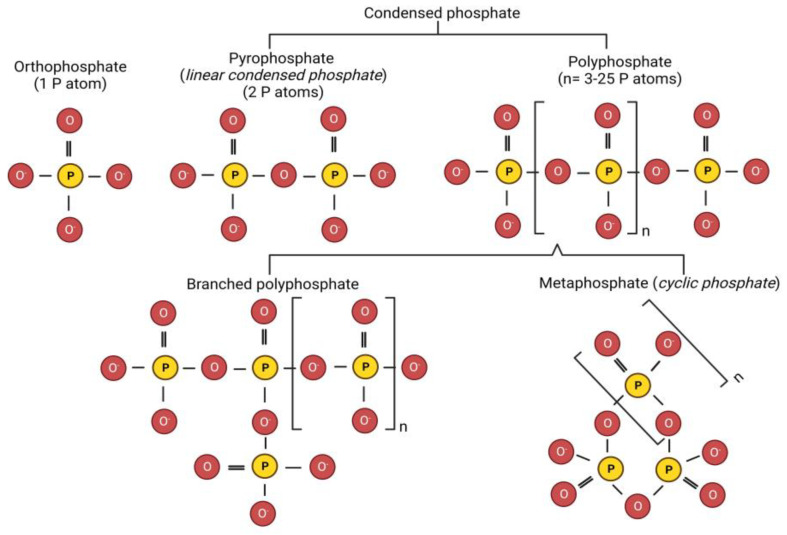
Molecular structure of different phosphate-based calcium sequestering salts used in processed cheese manufacture (P and O indicate phosphorus and oxygen atoms, respectively, whereas n represents the number of P atoms) (Created with BioRender.com, accessed on 13 February 2023).

Food-grade phosphate salts are derived by the purification of phosphoric acid (H_3_PO_4_). Based on the number of phosphate groups, phosphates are classified as orthophosphates (or orthophosphates) (1 P atom) and polymeric phosphates (multiple phosphates) (>1 P atoms) [22]. The basic structure of phosphates consists of each phosphorus atom surrounded tetrahedrally by four oxygen atoms. The molecular structure of different types of phosphate-based calcium sequestering salts are presented in Figure 1. Orthophosphates contain (PO_4_)^3−^ anions, which may have up to three oxygen atoms covalently bonded to other atoms. The oxygen atom may form linkages with phosphorus or other atoms (P-O-P), generating condensed phosphates (2 to ~25 P atoms). Linear condensed phosphates have one shared oxygen atom between neighboring PO_4_ groups, whereas metaphosphates (also termed as cyclic phosphates) have three or more oxygen atoms shared by neighboring PO_4_ groups. Under high temperature conditions, orthophosphates or longer chain phosphates with terminal -OH groups lose water (condensation reaction), thus bringing two phosphate molecules together to form polymeric phosphates [20]. Major phosphates used in processed cheese production are orthophosphate (e.g., disodium phosphate (P atom = 1)), linear condensed phosphates such as pyrophosphates (e.g., disodium pyrophosphates (P atom = 2)), and polyphosphates (P atoms = 3–25) (e.g., tripolyphosphate (3 P atoms)) [21].

## 3. Effects of Calcium Sequestering Salts on the Properties of Casein Micelles

### 3.1. Solubilization of Individual Caseins from Casein Micelles

The protein fraction of casein micelles is composed of four different casein fractions: α_s1_-, α_s2_-, β-, and κ-casein [5]. Calcium, both in colloidal and soluble form, plays a critical role in stabilizing the casein micelle structure. de Kort et al. [14] revealed the presence of “loosely bound calcium”, which is attached to negatively charged amino acid side chains and phosphate groups, and “strongly bound calcium” in the CCP complexes. Yamauchi et al. [23] defined approximately 40% of the colloidal calcium as “hard-to-exchange” calcium, which is associated with the colloidal phosphate of the casein micelles in milk. The removal of Ca^2+^ with CSS causes the dissociation and subsequent release of casein protein fractions from the micelles into the serum phase [1].

The dissociation of casein micelles by CSS is described as a cooperative process, which implies that the casein complex is either completely dissociated or largely intact; it does not imply that all casein micelles dissociate at the same time and same CSS concentration [1,24]. The dissociation of casein micelles is a two-stage process: a rapid first stage and a slow dissociation of the remaining casein complexes [1]. Firstly, the addition of low levels of EDTA disrupted the easily exchangeable Ca^2+^ bridges and dissociated the weakly held caseins (mainly α_s_- and β-casein) from the casein framework. Subsequently, the addition of more EDTA dissociated the colloidal calcium phosphate and Ca-α_s_-caseinate bonds and solubilized the framework protein (largely α_s_-casein [24]). With an initial decrease in Ca^2+^ ion activity, approximately 60% β-casein was dissociated from casein micelles without any micellar disintegration or decrease in the size of the casein micelles. The remaining β-casein (approximately 40%) seems to be strongly bound to α_s_- and κ-caseins, maintaining the structural framework of casein micelles [25,26].

Gaucher et al. [16] reported an order of solubilization of individual caseins (α_s1_ > β > α_s2_ and κ-casein) by potassium orthophosphate at levels up to 160 mM. The preferential solubilization of α_s1_- and β-casein by phosphate-based CSS has been linked to their greater Ca^2+^ binding ability due to their higher number of phosphoseryl residues in these caseins as compared to κ-casein [16]. The increased concentration of potassium phosphate from 20 to 160 mM in skim milk showed an increasing trend of α_s_- and β-casein amounts in a soluble fraction [16]. However, the ratio of individual soluble caseins was not affected by concentration and type of CSS (disodium uridine phosphate, DSP, TSC, sodium phytate, and SHMP) [14]. The casein fractions solubilized by CSS, as analysed in the supernatants of micellar casein isolates, were in the same ratio as that present in milk [14,18]. Pitkowski et al. [1] reported similar findings after the addition of EDTA and sodium polyphosphate. The whey protein β-lactoglobulin has been reported to remain unaffected by CSS, especially in samples without any heat treatment [16]. In heated MPC samples, CSS solubilized the κ-casein/whey protein aggregates, represented by the formation of high molecular weight (50–70 kDa) SDS-PAGE bands [27]. Lastly, the solubilization of casein is largely dependent on the ratio of CSS to casein, and results in the formation of small micellar particles, which changes with the increasing concentration of CSS.

### 3.2. Calcium Chelation Ability of Calcium Sequestring Salts

Calcium ions in casein micelles are part of the CCP complexes or attached to the phosphoserine, glutamate or aspartate residues. CSS competes with the inorganic phosphate of CCP and phosphoserine residues for the calcium ions. The different types of CSS have different affinities for calcium ions depending on which varying amount of CCP is released from the micelles [28]. The effectiveness of CSS to bind Ca has been evaluated mainly (i) by evaluating the Ca^2+^ ion activity using a calcium-ion selective electrode, (ii) by non-sedimentable Ca content after (ultra)centrifugation, or (iii) by soluble Ca content after ultrafiltration. A decreased Ca^2+^ ion activity and higher amount of non-sedimentable or soluble Ca is herein indicative of the higher calcium chelation ability of CSS [18].

The Ca binding ability of CSS exhibits the following order: long-chain phosphates > tri-polyphosphates > pyrophosphate (triphosphates and diphosphates) > citrate > orthophosphate [8,10]. SHMP and sodium phytate are strong CSS and bind Ca at a ratio of 1:3 and 1:6 (phosphate:Ca), respectively. DSP also has a strong calcium binding ability, but at a 3:2 ratio. Disodium uridine phosphate forms less strong complexes with Ca and binds Ca at a ratio of 1:1 [28]. EDTA is a stronger calcium binder than TSC [1,6], but is not permitted for use in most products. Calcium chelates preferentially with citrate compared to orthophosphate, because of the lower association constant of HPO_4_^2−^ (600 M^−1^) and H_2_PO_4-_ (10 M^−1^) than citrate (Cit^3−^) (10^5^ M^−1^) for calcium [4]. Furthermore, the effectiveness of TSC as a Ca sequestering salt was demonstrated by the reduced casein-bound Ca and casein-bound inorganic phosphate with increasing levels of added TSC to milk protein concentrate solution, and added citrate was not associated with casein [29]. Similarly, the structure of phosphate molecules influenced their calcium chelation ability instead of their organic and inorganic origin [28]. The replacement of the sodium cation of DSP and TSC with potassium showed no significant changes in calcium ion activity [12]. The influence of concentration of CSS, pH and temperature of the gel/solution on Ca^2+^ chelation is discussed in the following sub-sections.

#### 3.2.1. Effect of Calcium Sequestering Salt Concentration on Calcium Chelation Ability in Casein Micelle Suspensions

The majority of the published studies on casein micelle suspensions highlight increased calcium chelation with increasing concentration of CSS [1,15]. The calcium chelation ability of CSS becomes constant or shows very insignificant changes after reaching a certain level of CSS concentration [30]. For CSS such as DSP, TSC, SHMP, disodium uridine phosphate, and sodium phytate, Ca^2+^ ion activity decreased with the increasing concentration of CSS in micellar casein solutions (9%, *w*/*v*) within the concentration range of 15–60 mEq/L [18]. Since disodium uridine phosphate is a weak calcium chelator, only a slight decrease in Ca^2+^ ion activity was observed [14]. For phosphate-based CSS, at lower concentration, the added phosphate does not displace micellar calcium phosphate, since the affinity of Ca^2+^ is higher for micellar phosphate than for added phosphate. The addition of a higher phosphate level induces the displacement of micellar phase calcium, leading to the demineralization and solubilization of casein [6,16]. The level of Ca^2+^ chelation and solubilized casein levels were elevated by increasing the concentration of added EDTA from 5 to 50 mM/kg of skim milk. Moreover, the addition of 50 mM EDTA/kg skim milk disintegrated all of the casein micelles in the milk [6].

Culler et al. [30] modelled the decrease in the turbidity of skim milk by adding different CSS and defined C* as the threshold CSS concentration, at which rapid casein micelles dissociation occurs. SHMP showed the greatest decrease in turbidity at the lowest concentration of 0.33 mM, while monosodium phosphate and dipotassium phosphate had a C* of 278.22 mM and 216 mM, respectively. In case the polyphosphate concentration exceeds the critical level required for the complete dissociation of casein micelles, the radius of non-micellar casein particles is reduced [1].

#### 3.2.2. Effect of pH on Calcium Chelation Ability

The addition of CSS, acid or base to casein micelle containing dairy systems generates a pH change, which influences the Ca^2+^ sequestering ability of CSS. The addition of 150 mEq/L of sodium dihydrogen phosphate to milk protein concentrate dispersions (5%, *w*/*w*) decreased the pH from 6.9 to 6.2, whereas TSC caused a pH increase, and SHMP showed no changes in pH for 15–150 mEq/L [27]. The pH change created by added potassium dihydrogen phosphate to skim milk (pH 6.65 at 4.5 mM and 5.85 at 157 mM) induced the shift of acid-based equilibrium of the phosphate from HPO_4_^2−^ towards the H_2_PO_4_^−^ form. Due to the higher affinity for calcium of HPO_4_^2−^ (600 M^−1^) compared to H_2_PO_4_^−^ (10 M^−1^), the former is considered to be an effective Ca^2+^ binder [16]. DSP and TSC concentrations of 20 and 40 mM/L showed a decreasing influence on Ca^2+^ ion activity, with a pH change from 6.7 to 8.0. However, at very high concentrations (60–100 mM/L), non-significant influences of pH are reported [12].

In evaluating the effect of pH (5.0–8.8) on calcium chelation by ten different CSS, Culler et al. [30] reported the highest calcium chelation at pH 5.8 and 6.8. Similarly, with increasing pH from 6.7 to 7.3, the calcium ion activity of DSP, TSC and SHMP was found to decrease, but the decrease became smaller with increasing pH [18]. This is credited to more significant calcium phosphate complex formation and enhanced electrostatic repulsion between caseins at higher pH [31,32].

As pH values shift closer towards the isoelectric point of casein, a decrease in calcium chelation ability of CSS is also observed. Culler et al. [30] reported the lowest casein dispersion by different CSS at pH 5.0. Likewise, sodium phytate showed no significant binding of calcium below pH 5.0, but between pH 5.0 to 8.0, calcium binding occurs in the ratio of 6:1 (calcium:phytate) [33]. At very low pH values, especially near the isoelectric pH of casein (pH 4.6), the CCP and probably calcium-pyrophosphate complexes from TSPP might dissolve [3]. The casein micelle structure becomes more compact near the isoelectric pH. This tighter micelle structure is more dependent on aggregation from the protein charge than calcium phosphate bridges, reducing its susceptibility to dissociation caused by the calcium chelation of CSS [30].

#### 3.2.3. Effect of Heat Treatment on Calcium Chelation Ability

Heat treatment does not change the composition of aqueous solution of orthophosphate or orthophosphate solution in the presence of Ca [34]. At temperatures below 100 °C, hydrolysis of polyphosphate is almost negligible in water at pH 7 and pH 5.6; however, their composition is affected by the presence of Ca and temperatures above 120 °C [35,36]. The presence of Ca increased the hydrolysis of short-chain and long-chain polyphosphates (>4 phosphorus atoms) into trimetaphosphates and orthophosphates. This is linked to the increase in the positive charge of phosphorus atoms in long-chain phosphates by the presence of Ca, which may promote hydrolytic degradation [36]. Hydrolysis occurs through the nucleophilic reaction of water on the terminal phosphate unit leading to the breaking of the P-O-P bond and the formation of orthophosphates as end products [37].

The heating (>85 °C) of milk protein systems after adding CSS induces the rapid hydrolysis of linearly condensed phosphates to tripolyphosphates and pyrophosphates, and then more slowly to orthophosphates [9]. By heating micellar casein isolate solutions to 126 °C, SHMP hydrolyzes into sodium trimetaphosphate and sodium orthophosphate in acidic conditions, which induces a decrease in pH, the release of Ca ions, and increased calcium-ion activity. As a result, SHMP forms cross-links between caseins that are released during heating [18]. The hydrolysis of phosphate-based CSS increases the ratio of short-chain to long chain phosphates, thereby affecting the calcium chelation [9]. The heat treatment (128 °C for 5 min) of milk prior to the addition of sodium citrate and EDTA diminished the ease of solubilization of micellar calcium phosphate [6]. Generally, heating leads to the hydrolysis of phosphate-based CSS into short chain components, causing lower Ca chelation possibilities.

Overall, phosphates and citrates have very different effects on Ca chelation in simplified model milk protein systems, with each CSS demonstrating strong dependence on concentration, pH and temperature, as the role of CSS in a concentrated casein-based matrix may be more complex than previously believed. The influence of different calcium sequestering salts and their increasing concentration on the properties of casein micelles is presented in Table 2.

### 3.3. Casein Dispersion

The degree of casein dispersion within a processed cheese system is linked to the ability of CSS to bind calcium complexes and disrupt the calcium phosphate crosslinks in the para-casein matrix [3,7]. The flexible hydrophilic parts of caseins can be immobilized by calcium phosphate, which imparts a more rigid structure to casein micelles [40]. Casein dispersion has been evaluated by measuring the optical density/turbidity of model protein system (or processed cheese). Lower optical density or decreased turbidity represents more extensive casein dispersion or removal of CCP from the micelles [7,14]. The dissociation rate and extent is largely determined by the ratio of CSS to casein [1]. Panouillé et al. [2] and Pitkowski et al. [1] observed that calcium chelators induce the dissociation of intact casein micelles into smaller casein particles containing 10–15 casein proteins. A mixture of CSS (sodium polyphosphate and sodium citrate) dissociated casein micelles into smaller particles, with a diameter of 12 nm [2].

Based on the findings of different studies, phosphates with longer chain length and a higher proportion of polyphosphates in the CSS mixture showed higher casein dispersion [7,10]. de Kort et al. [14] and de Kort et al. [18] reported that casein micelles were dissociated in the order of disodium uridine phosphate < DSP < TSC < sodium phytate < SHMP. Casein micelles were not dispersed by the addition of DSP to a milk protein concentrate solution [15]. At a concentration of ≥45 mEq/L of SHMP, most of the casein micelles were dissociated, induced by pH decrease, the increased net negative charge of casein micelles, and the depletion of CCP from the casein micelles [18]. Similarly, the addition of 150 mEq/L of TSC to transglutaminase-treated skim milk removed all of the CCP from the casein micelles, while the micelle remained intact [5,41].

Overall, the casein dispersion ability of different CSS follows a trend similar to the Ca^2+^ sequestering ability of the CSS, but the ability of some CSS to cross-link caseins simultaneously creates differences in casein dispersion ability. SHMP and sodium phytate have six homogeneously distributed and twelve (clustered in pairs) negative charges around the molecule, respectively. This led to a stronger calcium sequestering ability of sodium phytate than of SHMP. The immediate strong chelation of Ca by sodium phytate may leave no charge or free calcium ions available for the cross-linking of caseins. Thus, sodium phytate does not cross-link caseins, whereas SHMP does, thereby contributing to higher casein dispersion by SHMP [14,28]. Similarly, tetrasodium pyrophosphate with four homogenously distributed charges around its molecule cross-links casein more easily than SHMP [3]. Ultimately, the commencement and degree of casein dispersion is largely dependent on the type and concentration of CSS, which could be helpful in controlling the viscosity, turbidity and heat stability of dairy systems. Furthermore, the insights on the effect of the mixtures of CSS on casein dispersion could provide new opportunities for modifying the desirable properties of casein-based matrices.

### 3.4. Formation of Complexes between Calcium Sequestering Salts and Calcium

Phosphate-based CSS may associate with dispersed caseins, while caseins dispersed by TSC may not necessarily aggregate and form gels [3]. Interestingly, TSC chelates Ca from indigenous CCP and forms soluble Ca citrate complexes, while DSP chelates Ca and forms an insoluble Ca phosphate complex, which may be trapped within the protein matrix [12]. EDTA also forms soluble complexes with Ca, which are less soluble than citrate-Ca complexes [1]. The tendency of TSC to form soluble complexes decreases the amount of casein-bound Ca and P. Instead, in the case of phosphate-based CSS, the formation of Ca phosphate complexes increases the casein bound Ca and P [15].

At a lower concentration (0.1%) of SHMP and TSPP, insoluble Ca-phosphate complexes were observed [29]. At a higher concentration of SHMP (0.5–0.7%), soluble CaHMP complexes were formed. This is attributed to the excessive charge repulsion resulting from the multiple negative charges introduced by SHMP molecules [15]. The addition of tetrasodium pyrophosphate dispersed the caseins and formed casein-calcium pyrophosphate complexes [29]. Shirashoji et al. [42] reported the formation of insoluble casein-calcium pyrophosphate complexes at higher concentrations of TSPP (>1%), with a portion of TSPP remaining in the soluble phase. The binding of calcium pyrophosphate complexes with dispersed casein could reduce the charge repulsion, thus facilitating the hydrophobic interactions between hydrophobic segments of caseins and promoting aggregation [3].

Condensed phosphates such as pyrophosphate, tripolyphosphate and hexametaphosphate form more stable casein-Ca-phosphate complexes than with orthophosphate. The addition of tetrasodium pyrophosphate (7.6 mM) to milk protein concentrate solution (51 g/L) dispersed the casein and formed casein-Ca pyrophosphate complexes [29]. The dispersion of casein is due to the loss of calcium phosphate cross-links, which may expose charged phosphoserine groups, thus increasing electrostatic repulsion between caseins [43]. The formation of stable casein-Ca-phosphate complexes could be attributed to complexes of condensed phosphates, with dispersed casein leading to a reduction in the electrostatic repulsion between casein molecules [3]. The stability constant (the equilibrium constant for complex formation, which measures the strength of interaction between reagents involved in complex formation), indicates the relative efficiency of chelators’ complexation with Ca^2+^ and is also higher for pyrophosphate (5.0) than citrate (3.5) [44]. Furthermore, in processed cheese systems, phosphates associated with casein micelles might act as cross-linking agents within or between casein micelles, thus affecting their functional attributes such as low meltability and higher firmness.

### 3.5. Casein Hydration

Rennet casein is insoluble in water owing to the presence of calcium mediated cross-bridges [17]. In order to add more water to the processed cheese, casein locked in the micellar structure has to be released by the usage of CSS [45]. Ca chelation by CSS swells and partially hydrates the insoluble casein and converts it to water soluble caseinate. This is achieved by the exchange of Ca^2+^ in the casein network with monovalent cations (e.g., Na^+^, H^+^, K^+^) of CSS, also leading to the increased negative charge on caseins [12,14]. Owing to calcium chelation, the concentration of free calcium ions decreased in the continuous phase, thereby increasing the negative charge and electrostatic repulsion of the casein micelles, finally leading to more hydrated and swollen casein micelles [14]. The extensive hydration of casein molecules permits their interaction with the oil phase, promoting the stabilization and emulsification of fat globules [45].

Cavalier-Salou et al. [38] suggested an increase in para-casein hydration with the increasing chain length of sodium phosphates added to cheese analogues. TSC gives lower casein hydration than ortho- and pyro-phosphates [39]. Huppertz et al. [41] also showed the swelling of casein micelles induced by trisodium citrate (0–50 mM) in cross-linked casein micelles suspension caused by the dissociation of micellar calcium phosphate. The addition of sodium citrate (238 mM) and sodium phosphate (173 mM) was linked to better rehydration rates and the higher moisture content of the native phosphocaseinate suspension. CSS solubilized the casein micelles, and water bound to micellar casein was more difficult to remove than water bound to soluble casein [46].

Sodium cations are more effective binders than potassium to carboxylate anions of amino acid residues in casein. The change of sodium cation with potassium in CSS leads to less cation binding and consequently less hydrogen ion displacement, eventually causing a higher pH. This higher pH leads to enhanced electrostatic repulsion between casein molecules, which may facilitate better protein hydration. It is also suggested that smaller hydrated potassium ion sizes facilitate higher calcium chelation and the easier hydration of the casein matrix [47]. A small variation in moisture content causes large changes in textural, rheological and functional attributes of processed cheese, especially at low moisture levels. Thus, the degree of casein hydration could have a significant role in achieving the maximum level of moisture content and the final desirable attributes of processed cheese.

## 4. Effect of Calcium Sequestering Salt on the Properties of Processed Cheese

The manufacturing of processed cheese involves the selection of different natural cheeses, the cleaning and size reduction of natural cheeses into small curd particles, the mixing with water and sequestering salts, the shearing of the blend under the influence of heat, and hot packaging and cooling. The conversion of milk into natural cheese involves the destabilization of the milk protein network, mostly calcium phosphate, into a concentrated para-casein network occluding fat [32]. The protein in natural cheese curd occurs as para-casein micelles fused in a network, which is rendered insoluble by inter-protein linkages mediated by calcium, colloidal calcium phosphate, and hydrophobic interactions between uncharged amino acid residues. On the conversion of natural cheese into processed cheese, the calcium phosphate para-casein network is deconstructed with partial protein solubilization, enabling it to bind water and emulsify free fat released during the heating and shearing stage [21]. This is confirmed by the increased level of water-soluble protein from 5–20% in natural cheese to 60–80% in processed cheese [48].

The “loose” oil-in-water (o/w) emulsion of natural cheese consisting of the concentrated gelled calcium phosphate para-casein network changes into a “finer” o/w emulsion in a concentrated casein(ate) dispersion in processed cheese. The hydrated casein/para-casein immobilizes free serum and emulsifies free fat into emulsified fat globules and creates a stabilized processed cheese. The fat globules in natural cheese are naturally emulsified by the native fat globule membrane consisting of protein and phospholipids, whereas in processed cheese, fat is emulsified with a reformed layer of re-hydrated para-caseinate. The fat globules in processed cheese are considered as pseudo-protein particles, which may interact with other emulsified fat globules [9]. Depending on the formulation, processing conditions and type of sequestering salts, the final processed cheese product may vary from firm and sliceable to soft and spreadable. However, differences in the functionality of CSS affects the properties of processed cheese and offers the ability to cheese manufacturers to customize the properties of the final product. CSS facilitates the processed cheese manufacturing process by sequestering calcium, enabling the swelling and hydrating of casein, casein peptisation, the emulsifying of free fat, the demineralising of casein, and pH adjustment [21]. A myriad of research studies have compared the effect of different types, combinations and levels of CSS on different physiochemical and functional properties of processed cheese. This section describes the influence of CSS on various physiochemical, functional, rheological, and sensory properties of processed cheese.

### 4.1. Emulsion Droplet Size

Caseins are the major emulsifiers in the processed cheese matrix, and a lower degree of emulsification is revealed by the larger diameter of fat globules [49]. The ability of CSS to promote emulsification generally coincides with the trend of calcium sequestration. The emulsification potential of CSS follows the following order: tripolyphoshates > pyrophosphates > polyphosphates > citrates ≈ orthophosphates ≈ sodium aluminium phosphates [9,38,39]. A higher degree of calcium sequestration causes more intensive casein dispersion developing the emulsification and hydration properties of these proteins. These proteins stabilize the fat globules by acting as a membrane leading to fat emulsification and higher casein cross-links [10].

During the manufacturing of processed cheese, free fat is separated during the initial heating stage, which is re-emulsified by CSS under the influence of heating [50]. Emulsified fat globules have a lower tendency to coalesce on reheating and are generally thermostable, contributing to the lower meltability of processed cheese [51]. Processed cheese samples prepared with polyphosphates and pyrophosphates had a higher number of fat globules compared to those with citrates and orthophosphates [50]. Low levels of emulsification results in softer processed cheese, and well emulsified processed cheese shows higher hardness and reduced meltability [52].

### 4.2. Textural Properties

In the course of processed cheese manufacturing, the addition of CSS in combination with heating and high-speed shearing disperses the insoluble casein matrix. During the cooling stage, these dispersed casein strands reassociate, besides CSS-Ca complexation, which significantly affect the textural properties of processed cheese [43]. A higher extent of casein dispersion in the processed cheese matrix increases the emulsifying and hydrating potential of caseins, which is responsible for the stabilisation of fat and water available in processed cheese. Concurrently, better protein hydration and fat emulsification generates a higher intensity of casein cross-links and a harder processed cheese [3,8].

#### 4.2.1. Effect of Calcium Sequestering Salt Type on Textural Attributes of Processed Cheese

Trisodium citrate provided a higher value of processed cheese hardness as compared to disodium phosphate and orthophosphates, but a lower value than polyphosphates and pyrophosphates [51,53]. These differences were mainly linked to the bigger fat globule size observed in DSP-based cheeses, which have low surface area and less interactions with protein [51]. During processing (melting stage), polyphosphates undergo rapid hydrolysis into triphosphates and diphosphates, causing a substantial increase in the hardening of processed cheese [52]. This is attributed to the higher ability of products of hydrolysis (especially triphosphates) to aggregate casein and emulsify fat, thus forming a 3-D network and leading to a more rigid and elastic processed cheese [9,29,52]. About 50% of the added phosphates are hydrolysed during the melting procedure, and the remainder is hydrolysed after 7 to 10 weeks of storage [54]. The products of hydrolysis possess a greater ability to induce aggregation by the formation of the caseinate-Ca phosphate complex and form a more rigid and elastic structure. The ability to form a rigid structure and support a three-dimensional network follows the following order: orthophosphate < polyphosphate < diphosphate < triphosphate [9,29]. Furthermore, diphosphates and triphosphates cause better fat emulsification, resulting in the higher firmness of processed cheese [50]. It has also been suggested that citrates dissociate on cooling post-manufacture, subsequently acting as a calcium ion source. This also means that some of the insoluble Ca-citrate present in processed cheese can dissociate/dissolve on cooling. These calcium ions cross-link the CSS anions attached to casein and increase the hardness of processed cheese [13,51,55].

Within the phosphate CSS category, the hardness of processed cheese generally increases with the increasing number of phosphorus atoms present in CSS [56]. Orthophosphates are low molecular weight substances with the ability to permeate among cross-linked caseins and strongly bind water. In addition, orthophosphates have very low calcium ion exchange ability, resulting in the lowest hardness of processed cheese [7,52]. Diphosphates act as cross-linking agents by forming complexes with calcium ions (caseinate-Ca phosphate complexes) and reduce charge repulsion, thus inducing the gel formation of casein proteins and higher hardness values [55,56]. The excessive incorporation of diphosphates in processed cheese binds too much calcium, making it unavailable for diphosphate-calcium cross-linking interactions, and resulting in the lower hardness of processed cheese [56].

Long chain polyphosphates can not only bind calcium strongly, but also disperse casein effectively. Polyphosphates bind to casein fractions and provide them with strong multiple negative charges [10]. These extensively charged casein fractions disallow sufficient gel formation, thus generating difficulties in casein re-association through hydrophobic segments [3,8]. In phosphate based emulsifying salts, ion exchange ability (Na^+^ for Ca^2+^) increases with the increasing number of phosphorus atoms linearly bound in a phosphate molecule. Long-chain polyphosphates have the highest ion-exchange ability, resulting in the greatest degree of hardness of processed cheese. When polyphosphates are used in combination with orthophosphate and/or diphosphate up to 50–60%, greater hardness is observed. In the case of a higher proportion of polyphosphates (60% or more) in CSS mix, the hardness of processed cheese is decreased [52,56]. This could be explained by the ability of polyphosphates to give caseins a multiple negative charge, which reduces the specific effects of orthophosphates and diphosphates, and the formation of a three-dimensional network of the melt dominates. As a result, the ability of long-chain polyphosphates to strongly disperse casein chains prevail, leading to increased casein hydration and better fat emulsification. Thus, orthophosphates and/or diphosphates in a lower amount cannot show their specific properties, and yields processed cheese with a lower hardness [57].

The use of SHMP imparted higher hardness to processed cheese in comparison to TSC or orthophosphates. This is attributable to the enhanced casein dispersion (hydration, peptization, or swelling) and the Ca chelation ability of SHMP. SHMP disperses the casein molecules, which results in greater cross-linking during the cooling stage of processed cheese and a firmer cheese [8]. Another study reported highest hardness of SHMP incorporated processed cheese followed by tetrasodium pyrophosphate, trisodium citrate and disodium phosphate cheese, respectively [53]. However, processed cheese prepared with tetrasodium pyrophosphate (TSPP) had higher hardness in comparison to DSP and SHMP, which was linked with the higher fat particle size reduction ability of TSPP. Smaller fat globules offer a higher number of interaction points with protein, thus making the network firmer [13].

#### 4.2.2. Effect of Calcium Sequestering Salt Concentration on Textural Attributes of Processed Cheese

Generally, increasing the concentration of CSS has been linked with the increasing hardness of processed cheese. An increase in the level of TSC, TSPP, STPP, SHMP and DSP from 1 to 3% showed an increase in hardness values [13]. Shirashoji et al. [51] also reported the increased hardness of processed cheese with the increasing concentration of TSC from 0.25% to 0.75%. The increased concentration of CSS improves fat emulsification and casein dispersion. Obviously, at a lower CSS concentration, fat globules are poorly emulsified [55]. With increased CSS concentration coupled with shearing treatment during the cooking stage of processed cheese manufacturing, fat globules are emulsified and covered as casein. These casein-covered fat globules behave like large pseudo-protein particles and are actively incorporated into the casein network of processed cheese. This allows the formation of a more reinforced network of protein stabilized fat globules and the higher hardness of processed cheese [9].

Overall, several researchers present conflicting findings for textural attributes of phosphate- and citrate-based processed cheese. These discrepancies are related to the differences in blend formulation, processing conditions, pH, and type and concentration of CSS. The effect of different CSS on physico-chemical, textural, functional and sensorial attributes of processed cheese are summarized in Table 3.

### 4.3. Viscoelastic Properties

The viscoelastic properties of processed cheese have been evaluated and described by mainly performing two different oscillatory rheological tests, i.e., temperature sweep and frequency sweep. During temperature sweep, the storage or elastic modulus (G′) and loss or viscous modulus (G″) are measured at a fixed frequency and a specified rate of temperature increase. A sample may show liquid-like behavior (G″ > G′), solid-like behavior (G″ < G′), and point of heat-induced state (solid ↔ liquid) transition (G″ = G′). The ratio of G″ to G′ is termed as the phase angle (δ), and a phase angle equal to 45° (tan δ = 1) represents the gel-sol transition point, while δ greater than 45° indicates a liquid-like (or melt) behavior (Lee & Anema, 2009). The breakage of intermolecular casein interactions by CSS creates the potential for new interactions between caseins via hydrogen, hydrophobic, and electrostatic bonds [51]. Lucey et al. [43] reported that lower values of the loss tangent for processed cheese indicates less bond mobility.

The calcium sequestration leads to the swelling of casein micelles, followed by their enhanced hydration and voluminosity, which in turn increases the viscous modulus [12]. Processed cheese samples prepared with DSP and TSC exhibited more liquid like behavior, and TSPP and pentasodium tripolyphosphate based processed cheese showed solid-like behavior [53]. The formation of a more rigid structure (higher G′) of processed cheese by different CSS showed the following order: orthophosphate < polyphosphate < diphosphate < triphosphate. Processed cheese with a higher number of phosphate groups permits interactions within or between casein molecules, especially via calcium bridges resulting in a more rigid structure [52]. The increasing concentration of DSP from 0.75 to 3.40 g/100 g processed cheese reduced the maximum tan δ and increased G′ (Guinee & O’Kennedy, 2012). Shirashoji et al. [51] and Shirashoji et al. [8] publicized similar trends for trisodium citrate and sodium hexametaphosphate at added levels of 0.25–2.75 g/100 g, respectively. The textural and rheological properties of processed cheese are largely dependent on similar factors and show a similar kind of variation. It would be interesting to study the correlation among different measurable variables defining these properties.

### 4.4. Melting Properties

Meltability is the physical characteristic of cheese highlighting the melting properties of fat globules and the reorganisation of the protein structure (the major weakening of the bond between proteins) [52]. Tatsumi et al. [64] determined a decrease in meltability of processed cheese with an increase in insoluble casein, determined by the centrifugation of model cheese dispersions containing sodium caseinate, butter fat, and water. However, the studied model’s processed cheese systems did not contain sequestering salts, but the decrease in meltability was linked to the formation of insoluble casein at 80 °C due to the heat-induced aggregation of casein. When the number of casein-casein interactions decreases due to the extensive proteolysis of natural cheese or the chelation of Ca, the meltability of the processed cheese is increased. The reduced number of colloidal calcium phosphate cross-links and increased electrostatic repulsions by exposure of negative charges of phosphoserine residues increased the meltability of processed cheese added with TSC [43]. The addition of disodium oxalate to the CSS mix for rennet casein-based processed cheese considerably improved the meltability than that prepared with disodium phosphate and tetrasodium pyrophosphate individually. Oxalate not only acted as a calcium sequestering agent but also reduced the emulsification, which was indicated by the size of the large fat globules [58].

The TSC-incorporated model rennet casein-based processed cheese showed higher meltability than that prepared with DSP, tetrasodium pyrophosphate, and sodium aluminium phosphate, owing to the greater casein dispersion by TSC. Phosphate-based CSS demonstrates extra casein-CSS interactions, reducing the melt [58]. The melting properties of processed cheese prepared with different CSS shows the following trend: sodium aluminium phosphate ≈ trisodium citrate > disodium phosphate > sodium tripolyphosphates ≈ tetrasodium pyrophosphate > long chain polyphosphates [9]. With the increasing concentration of disodium phosphate from 0.5 to 4.0 g/100 g processed cheese, a decreased trend of meltability was reported [48]. Similarly, the highest concentration of TSC at the level of 2.75% exhibited the lowest meltability in processed cheese [51]. The reduction in meltability was due to increased fat emulsification and greater immobilisation and structuring of the aqueous phase [48]. A higher degree of fat emulsification produces a higher number of casein-covered emulsified fat particles, positively contributing to the structural integrity of the processed cheese matrix [13,48]. Overall, the hardness and meltability of processed cheese seem to be inversely related. General trends emerge showing the desirable melting properties of orthophosphates, citrates and sodium aluminium phosphates. In contrast, condensed phosphates have poor melting properties.

### 4.5. pH

The pH of processed cheese commonly varies between 5.5 and 6.0, depending on compositional and processing factors. High pH processed cheese products are moist and elastic, while a low pH leads to dry, short (more brittle) and crumbly processed cheese with a high susceptibility to fat separation (correlated with less fat emulsification) [61,65]. At a pH of 5.2, granular processed cheese with less emulsification and large protein aggregates was obtained [65]. CSS plays a significant role in the final pH adjustment and stabilization of processed cheese owing to its buffering capacity [21,56]. The pH of 1% aqueous solution of a few commonly used CSS such as trisodium citrate, disodium phosphate, tetrasodium pyrophosphate, and sodium hexametaphosphate was found to be 6.2–6.3, 8.9–9.1, 10.2–10.4, and 6.0–7.5, respectively [21]. The pH of processed cheese (without adjustment) increased in the order of sodium polyphosphate (5.8) < disodium phosphate (6.6) < sodium tripolyphosphate (6.7) < tetrasodium pyrophosphate (6.8) < trisodium phosphate (6.9) [52].

It has been established that sodium salts of phosphates increase the pH of processed cheese in all but a few exceptions, such as disodium pyrophosphate at the rate of 3 g/100 g processed cheese, which yielded a pH of 4.7 in the final product [52]. A few other examples of acidic CSS are monosodium citrate, monosodium phosphate, and sodium hexametaphosphate, which resulted in processed cheese with a pH of 5.2 or lower, having mealy, dry and crumbly textures [61]. The pH of the processed cheese samples decreased with the increasing amount of polyphosphates ((NaPO_3_)_n_, where n was 15–20)) in the ternary mixture of CSS [56]. Nagyová et al. [10] reported a decrease in pH of processed cheese with the increase in length of the phosphate chain of CSS. This was credited to the higher availability and release of hydrogen cations in long phosphates as compared to shorter phosphate CSS. Owing to the acidic pH of monosodium citrate (pH of 1% aqueous solution—3.75) and disodium citrate, these are suggested to achieve the desired pH of processed cheese, when high-pH cheese, skim milk solids, or mature natural cheese are used in the processed cheese blend [9].

### 4.6. Color

The color of processed cheese is largely dependent on the type, composition and properties of natural cheese, processing parameters, colorants (majorly annatto and paprika), and other ingredients. The color of processed cheese usually varies from yellow to orange; in some circumstances where mould cheeses are used it could have a blue, green or greyish tinge [66]. Fat content and the size of the fat globules dispersed in the cheese matrix also influence the color of the processed cheese. Smaller fat globules disperse more light, leading to a whiter color of processed cheese [59]. The lightness values (L*) decrease upon the addition of CSS due to Ca binding and the dissociation of casein micelles. The addition of sodium phosphate to milk protein concentrate dispersions showed no change in L* values, indicating no dissociation of casein micelles. Since SHMP also binds colloidal calcium and dissociates casein micelles, SHMP showed a higher decrease in L* values than TSC within the similar concentration range of 15–150 mEq/L [27]. With increasing pyrophosphate content, shinier and whiter processed cheese samples were obtained due to their higher soluble protein content [60]. Processed cheese prepared with SHMP was whiter than TSC and tetrasodium pyrophosphate, which was linked with the smaller fat globule size of SHMP and the absence of interactions between citrate and casein in TSC-based processed cheese [59].

### 4.7. Sensory Properties

There are very few studies pertaining to the influence of CSS on the sensory evaluation of processed cheese. The replacement of sodium-based CSS by potassium-based CSS up to 50% showed no adverse influence on the sensory qualities of processed cheese [62]. The replacement with potassium-based CSS above 50% led to a certain salty flavor and metallic or bitter taste [63]. A soapy, chemical or salty flavor has been associated with phosphates. At a level of 2% (*w*/*w*), pyrophosphates may impart bitterness in processed cheese [61]. Sodium citrates generally impart a “clean” flavor to processed cheese, whereas potassium citrate may cause bitterness [9]. The use of sodium potassium tartrate caused grittiness due to the formation of calcium tartrate [61].

## 5. Conclusions

The influence of a large number of variables (processing as well as compositional) on the rheological, functional and textural attributes of processed cheese manifests that it is a challenge to have a single method and set of processing conditions. To better control processed cheese quality, there needs to be a better understanding of ingredient functionality that influences the possible chemical interactions responsible for its quality. The effect of CSS on pH, calcium chelation, the degree of casein dissociation, and fat emulsification is interlinked and greatly explains the variations in processed cheese quality attributes.

## Figures and Tables

**Table 1:** Permitted calcium sequestering salts in processed cheese as per code of federal regulations 21CFR133.169 [9,11].

Group	Permitted Calcium Sequestering Salts	Chemical Formula	Molecular Weight (g/mol)
Citrate	Sodium citrate	C_6_H_5_Na_3_O_7_	258.07
	Potassium citrate	C_6_H_5_K_3_O_7_	306.39
	Calcium citrate	C_12_H_10_Ca_3_O_14_	498.40
Orthophosphate	Monosodium phosphate	NaH_2_PO_4_	119.97
	Disodium phosphate	Na_2_HPO_4_	141.96
	Trisodium phosphate	Na_3_PO_4_	163.94
Pyrophosphate	Tetrasodium pyrophosphate	Na_4_P_2_O_7_	265.90
	Sodium acid pyrophosphate	Na_2_H_2_P_2_O_7_	221.94
Polyphosphate	Sodium hexametaphosphate	Na_6_O_18_P_6_	611.77
Aluminum phosphate	Sodium aluminum phosphate	AlNaO_4_P^+^	144.94
Potassium based CSS	Dipotassium phosphate	K_2_HPO_4_	174.18
	Sodium potassium tartrate	C_4_H_4_KNaO_6_	210.16
Tartrate	Sodium tartrate	C_4_H_4_Na_2_O_6_	194.05

**Table 2:** Effect of different calcium sequestering salts on the properties of casein micelles.

Properties	Effect of Calcium Sequestering Salts (CSS)	Increasing CSS Level	Reference
Solubilization of individual casein fractions	Order of solubilization- αs1 > β > αs2 and κ-casein Ratio of individual soluble caseins is not affected by type of CSS	↑	[14,16]
Calcium chelation ability	Long-chain phosphates > tri-polyphosphates > pyrophosphate (triphosphates and di-phosphates) > citrate > orthophosphate	↑	[8,10]
Casein dispersion	Disodium uridine phosphate < disodium phosphate < trisodium citrate < sodium phytate < sodium hexametaphosphate	↑	[14,18]
Emulsion droplet size	Tripolyphoshates > pyrophosphates > polyphosphates > citrates ≈ orthophosphates ≈ sodium aluminium phosphates	-	[9,38,39]
Casein hydration	Phosphates: Increased casein hydration with increasing chain length of sodium phosphates Citrates: Lower than ortho- and pyro-phosphates	↑	[38,39]
Complex between CSS and Ca	Phosphates: Insoluble Ca phosphate complexes Citrates: Soluble Ca citrate complexes	-	[1,12]

The general effects of different calcium sequestering salts are summarized from the published literature. However, the precise effects of changing them may depend on several factors, including their interactions.

**Table 3:** Effect of different calcium sequestering salts on physico-chemical, functional and sensorial properties of processed cheese.

Properties	Effect of Calcium Sequestering Salts	Reference
Hardness	Strong effect of concentration and type of CSS- lot of contradictory reports	[51,52,53]
Viscoelastic	Less rigid structure (lower G′) of orthophosphate < polyphosphate < diphosphate < triphosphate.	[51,52]
Melting ability	Higher meltability of TSC-based processed cheese than DSP, tetrasodium pyrophosphate, and sodium aluminium phosphate Sodium aluminium phosphate ≈ trisodium citrate > disodium phosphate > sodium tripolyphosphates ≈ tetrasodium pyrophosphate > long chain polyphosphates	[9,58]
Color	Increasing pyrophosphate content: shinier and whiter processed cheese Whiter processed cheese with SHMP than TSC and tetrasodium pyrophosphate	[59,60]
pH	Sodium polyphosphate (5.8) < disodium phosphate (6.6) < sodium tripolyphosphate (6.7) < tetrasodium pyrophosphate (6.8) < trisodium phosphate (6.9) Decrease in pH of processed cheese with increase in length of phosphate chain of CSS	[52,56]
Sensory	Phosphates: Soapy, chemical or salty flavour Pyrophosphates: Bitterness at 2% (*w*/*w*) level Potassium based CSS: salty, metallic or bitter taste	[61,62,63]

## Data Availability

Not applicable.
